# Correction to: Cigarette smoke exposed airway epithelial cells-derived EVs promote pro-inflammatory macrophage activation in alpha-1 antitrypsin deficiency

**DOI:** 10.1186/s12931-023-02571-7

**Published:** 2023-11-04

**Authors:** Nazli Khodayari, Regina Oshins, Borna Mehrad, Jorge E Lascano, Xiao Qiang, Jesse R West, L Shannon Holliday, Jungnam Lee, Gayle Wiesemann, Soroush Eydgahi, Mark Brantly

**Affiliations:** 1 Division of Pulmonary, Critical Care, and Sleep Medicine, College of Medicine, University of Florida, 1600 SW Archer Rd Rm M453A, Gainesville, FL 32610 USA; 2 MilliporeSigma, Burlington, MO USA; 3 Department of Orthodontics, College of Dentistry, University of Florida, Gainesville, FL USA; 4 College of Medicine, University of Florida, 1600 SW Archer Rd Rm M453A, 32610 Gainesville, FL USA

**Correction to: Khodayari et al. Respiratory Research (2022) Sep 6;23(1):232**

10.1186/s12931-022-02161-z

After the publication of this original article [1], the authors identified an error in Fig. 3B. The images of GAPDH control was mistakenly placed within the control group into the CS group. The authors confirm that these corrections do not change the result interpretation or conclusions of the article and apologise for this error

The correct version of figure is given below.

**Fig. 3:**
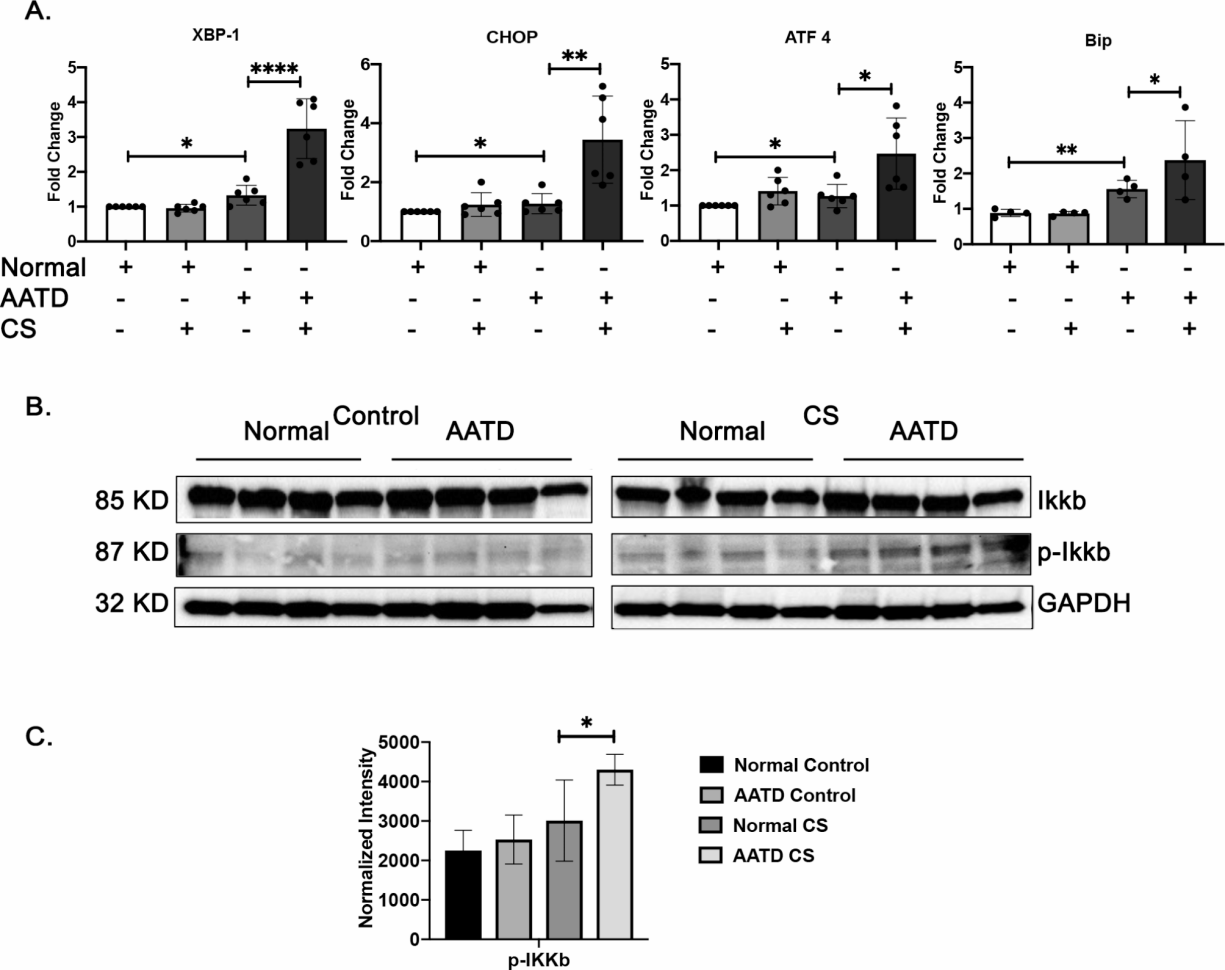
Induction of ER stress and NF-?B inflammatory pathway in AATD macrophages in response to cigarette smoke. **A** Comparison of the mRNA expression levels of XBP-1, CHOP, ATF 4, and BiP in normal and AATD macrophages exposed to air or cigarette. **B** Western blot analysis of 4 different normal and AATD macrophages incubated with control or CS exposed showing activation of NF-?B pathway in AATD macrophages after CS exposure. **C** Bar graphs showing the results of quantification and normalization of band intensities, *p < 0.05, **p < 0.005, ****p < 0.00005

