# Supplementary figures and images for: Cigarette smoke exposed airway epithelial cell-derived EVs promote pro-inflammatory macrophage activation in alpha-1 antitrypsin deficiency
Source: Respir Res. 2022 Sep 6;23:232. doi: 10.1186/s12931-022-02161-z (PMC9446525; doi:10.1186/s12931-022-02161-z)

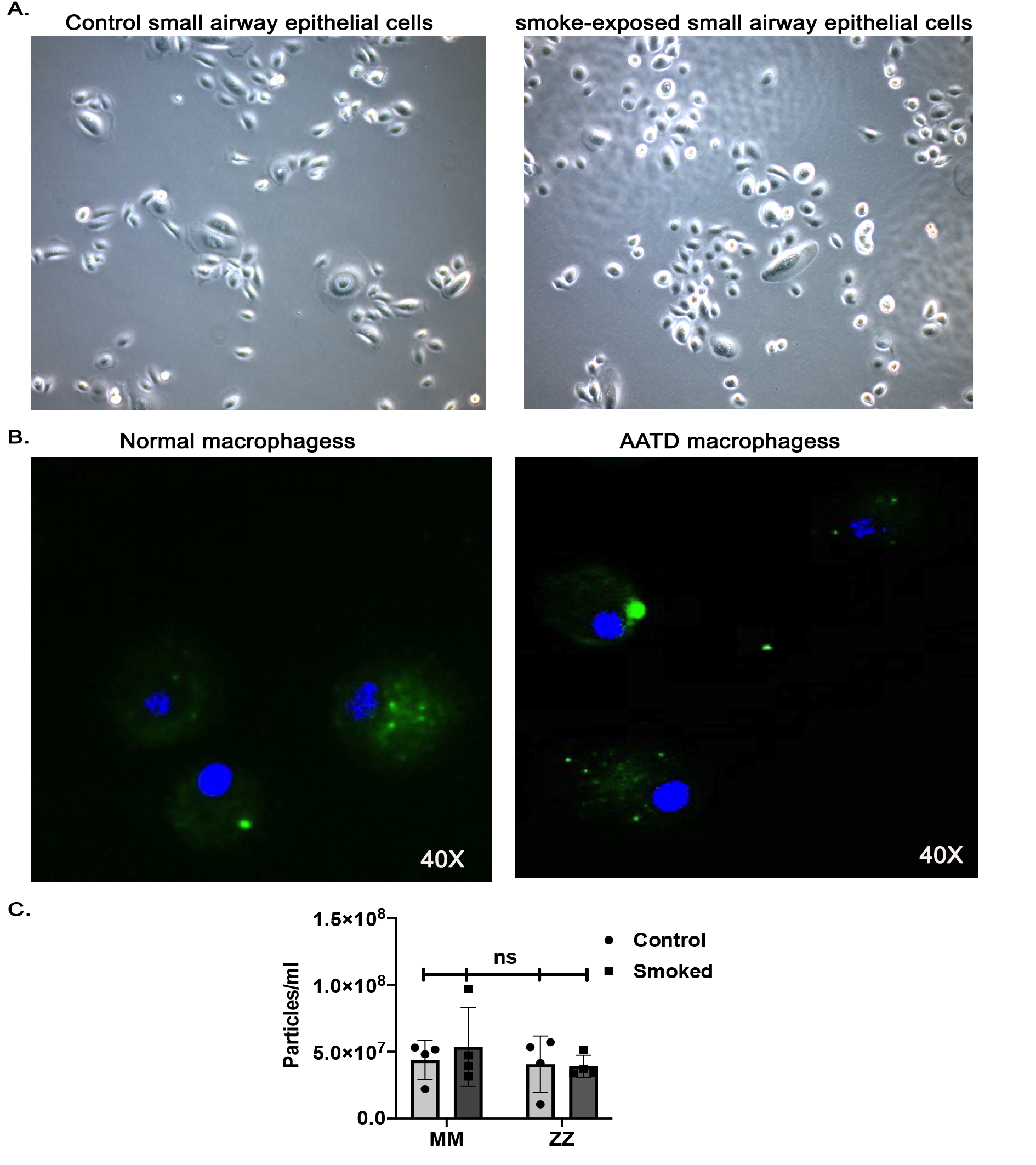

Supplement: Supplementary file 1 — Additional file 1: Figure S1. (A) The morphology of small airway epithelial cells before and after smoke was monitored using light microscopy. (B) DIO labeled (green) exosomes were incubated with normal and AATD macrophages for 1 h. The immunofluorescence pictures of trypsin treated cells shows green labeled exosomes inside the normal and AATD macrophages. (C) The concentration of EVs release by control and smoked normal (MM) and AATD (ZZ) macrophages as determined by Nano Track Analysis. [file 12931_2022_2161_MOESM1_ESM.tif]

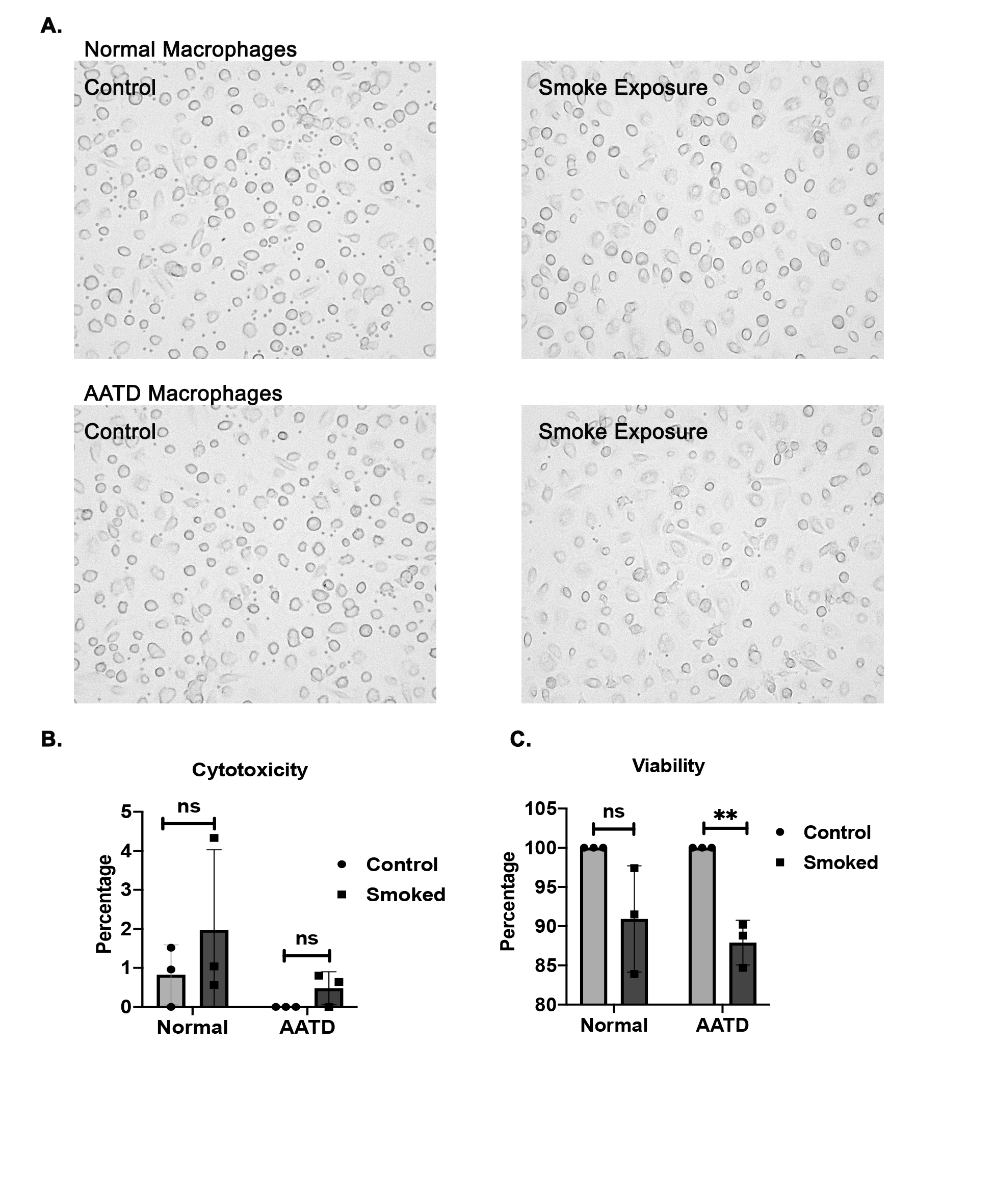

Supplement: Supplementary file 2 — Additional file 2: Figure S2. (A) The morphology of normal and AATD macrophages before and after smoke was monitored using light microscopy. (B) LDH assay indicating the percentage of cytotoxicity and (C) MTT assay indicating viability of normal and AATD macrophages have been presented. [file 12931_2022_2161_MOESM2_ESM.tif]

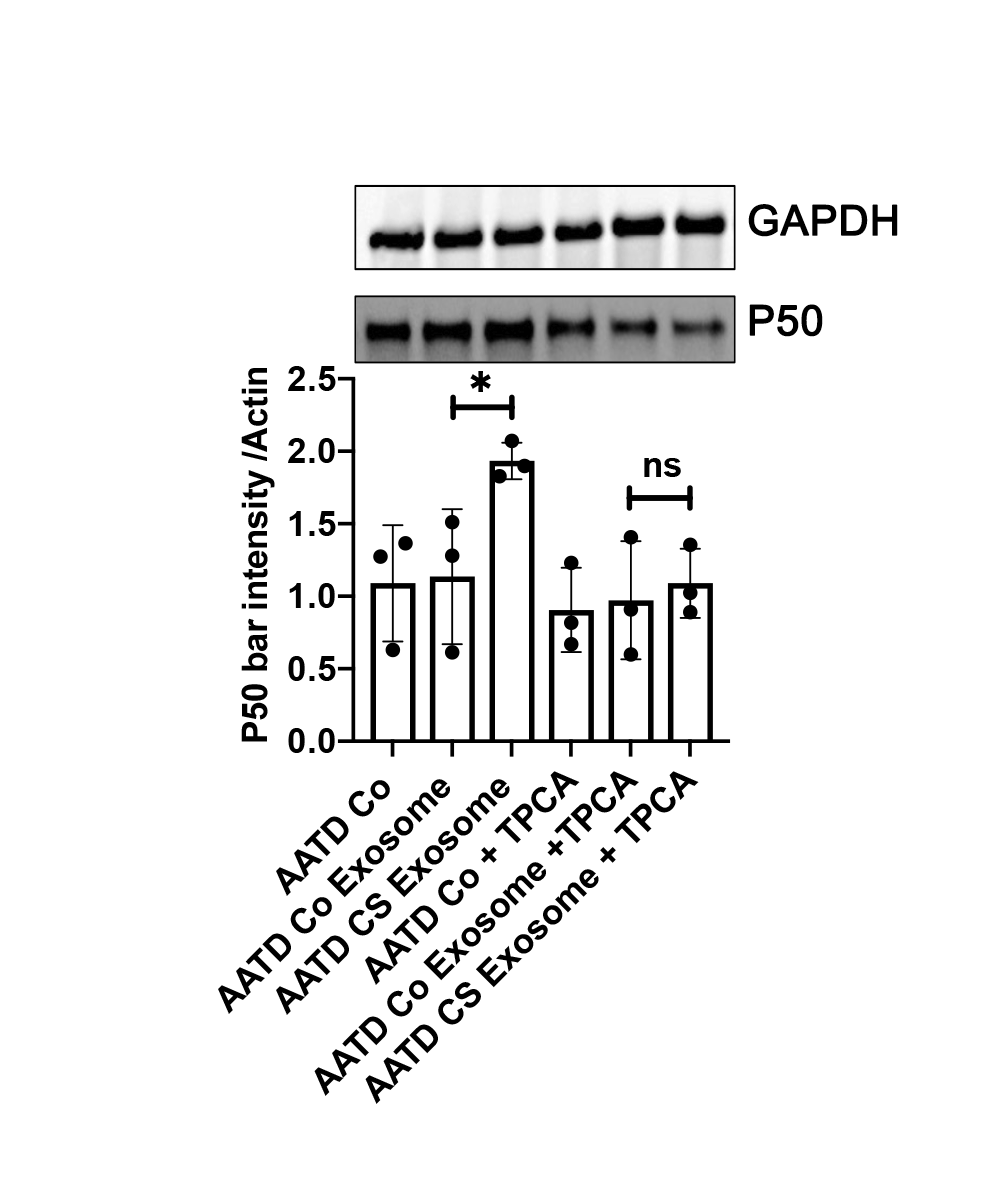

Supplement: Supplementary file 3 — Additional file 3: Figure S3. The representative blot of protein levels of P50 in AATD macrophages with/without NF-κB inhibitor (TPCA) accompanied with normalized bar intensities to actin (n = 3). [file 12931_2022_2161_MOESM3_ESM.tif]
